# Efficacy and Adverse Effects of Atropine for Myopia Control in Children: A Meta-Analysis of Randomised Controlled Trials

**DOI:** 10.1155/2021/4274572

**Published:** 2021-12-10

**Authors:** ChunWen Chen, JingYan Yao

**Affiliations:** Department of Ophthalmology, The First Affiliated Hospital of Soochow University, Suzhou, China

## Abstract

**Objectives:**

To explore the rebound effects and safety of atropine on accommodation amplitude in slowing myopia progression.

**Methods:**

We conducted a meta-analysis to testify proper dosage of atropine in children with myopia. We searched in PubMed, EMBASE, Ovid, and the Cochrane Library up to March 30, 2021. We selected randomised controlled trials (RCTs) that evaluated the efficacy of atropine for controlling myopia progression in children. We performed the inverse variance random-effects model to pool the data using mean difference (MD) for continuous variables. Statistical heterogeneity was assessed using the I^2^ test. Additionally, we conducted subgroup analyses and sensitivity analyses.

**Results:**

Seventeen RCTs involving 2955 participants were included. Myopia progression was significantly less in the atropine group than that of the control group, with MD = 0.38 D per year (95% confidence interval, 0.20 to 0.56). Less axial elongation was shown with MD = −0.19 mm per year (95% CI, −0.25 to −0.12). There was a statistically difference among various doses (*p*=0.00001). In addition, 1.0% atropine showed the rebound effect with MD = −0.54 D per year (95% CI, −0.81 to −0.26) and was more effective in the latter six months than in the former one. Less accommodation amplitude was shown in 0.01% atropine.

**Conclusion:**

The efficacy of atropine is dose dependent, and 0.01% atropine may be the optimal dose in slowing myopia progression in children with no accommodation dysfunction. A rebound effect is more prominent in high-dose atropine in the former cessation after discontinuation.

## 1. Introduction

Myopia, known as nearsightedness, is widely recognized as an urgent public health issue causing significant visual loss for a range of ocular comorbidities including cataract, retinal detachment, and glaucoma [1–3]. The high prevalence has been reported to be 80–90% in school children in certain East Asian areas in the past few decades [4–6]. The worldwide prevalence of myopia and high myopia is estimated to increase affecting nearly five and one billion people globally, respectively, by the year 2050 [7]. This silent epidemic should not be ignored [8]. Furthermore, early-onset myopic children are always accompanied with high progression rates and a higher incidence of high myopia [9, 10]. Thus, it is instant to prevent myopia promptly. The currently considered therapies for myopia include optical correction including bifocal spectacle lenses, orthokeratology lenses, multifocal contact lenses, additional time spent outdoor, and pharmaceutical agents such as topical atropine [6, 11, 12].

Atropine (low dose, 0.01%; moderate dose, 0.01% to 0.5%; and high dose, 1%) has been used to control myopia progression for many years [13–15]. The exact mechanism of how atropine retards is still unclear that may alter corneal curvature, vitreous chamber depth, lens thickness (LT), and anterior chamber depth (ACD) [16, 17]. The low-dose atropine has minimal influence on pupil size, loss of accommodation, and near vision for the prevention of myopia progression [14]. Patients using atropine may experience blurred vision, glare, photophobia, and allergic reactions [18]. In the recent findings, 0.05% atropine seemed to be the most effective dosage in myopia prevention [18]. One study demonstrated that atropine also caused a reduced myopic progression and rebound effect, which was less pronounced with lower dosage [19].

However, previous systematic review and meta-analysis have identified the efficacy and safety of atropine with ambiguous findings [20–23]. It was shown that the optimal dose of atropine may be 0.5% and 1% by Song et al. [21] and equally beneficial by Gong et al. [20], 0.01% by Zhao et al. [22], and 0.05% by Zhao et al. [23], but lacking consensus on atropine dose. As new clinical trials continually emerge, it is essential to conduct a meta-analysis to identify the optimal dose of atropine. The purpose of this study was to investigate the efficacy and rebound effects of different concentrations after its cessation and compare the rate of progression earlier and later during the first year. Moreover, the present analysis evaluated various doses of atropine with primary adverse effects on accommodation and ACD. Other adverse effects of atropine were analyzed including best-corrected visual acuity (BCVA), near vision, pupil size, intraocular pressure (IOP), tear break-up time (T-BUT), and LT.

## 2. Methods

This was a meta-analysis of existing RCTs; thus, approval by the institutional review board was not required.

### 2.1. Data Sources and Literature Searches

We searched PubMed, EMBASE, Ovid, and the Cochrane Library for RCTs in any languages to yield relevant studies from their inception to March 30, 2021. We used the following as key words: myopia, refractive errors, and atropine, as well as some relevant free terms. We also screened in the World Health Organization International Clinical Trials Registry Platform and ClinicalTrials.gov to retrieve additional ongoing trials. We used a protocol for the present review registered in the PROSPERO database (CRD42021247893).

### 2.2. Study Selection

We included only RCTs according to the following criteria: (1) participants were younger than 18 years with myopia; (2) atropine was used for at least one treatment arm, and (3) the study reported at least one outcome of interest, including the mean myopia progression (D per year), axial elongation, pupil size, accommodation amplitude, and any adverse effects. The exclusion criteria were as follows: (1) secondary articles such as review articles; (2) original data could not be extracted and obtained after contacting the author.

### 2.3. Data Collection

Two reviewers (Chun-Wen Chen and Jing-Yan Yao) independently screened data. In case of more than one data report from the same study, we included only the latest report to avoid duplicate counting of the data. Data from all doses were recognized as unique clinical trials. We conducted a focused discussion to resolve any disagreements. We extracted the following information from each trial: (1) study characteristics (author, year of publication, country, intervention and control group, and follow-up duration); (2) patient characteristics (the number of cases and age and baseline refraction).

### 2.4. Risk of Bias Assessment

We assessed the risk for bias of RCTs for the following six aspects according to the Cochrane Collaboration: random sequence generation, allocation concealment, blinding of patients and personnel, masking of outcome assessors, incomplete outcome data, and selective outcome reporting. We graded each of the item domains at “low,” “high,” or“unclear” risk of bias.

### 2.5. Statistical Analysis

We conducted analyses for changes in different concentrations of atropine versus control conditions based on RCTs. We calculated mean difference (MD) and 95% confidence intervals (CIs). We assessed heterogeneity with the I^2^ statistic. Also, I^2^ value greater than 50% indicates substantial heterogeneity. Subsequently, we considered performing sensitivity and subgroup analyses to investigate the source of heterogeneity. We performed direct comparisons using Review Manager (version 5.3; Cochrane Collaboration). For all comparisons, the stated values represent differences in final outcome between the intervention and control group. In terms of refractive diopters, a positive MD indicates that the intervention is better (less myopia progression). In terms of axial length, a negative MD indicates the intervention is better (less axial elongation). *p* value was thought to be significantly meaningful if less than 0.01.

## 3. Results

### 3.1. Study Characteristics

**Figure 1:**
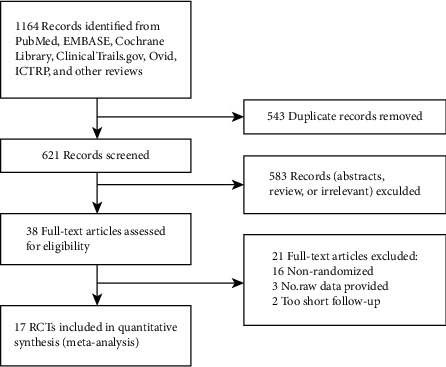
Flowchart for the study analysis. ICTRP = International Clinical Trials Registry Platform; RCTs = randomized controlled trials.

We identified 1163 studies through literature searches, and the remaining 45 full-text articles and, ultimately, 17 RCTs [13, 15, 16, 23–36] constituted the data for analysis (Figure 1). A total of 2955 participants were included comprising 1584 and 1371 in the intervention and control groups. The characteristics of the included studies are described in Table 1. Low-dose atropine (0.01%) was reviewed in eight studies [13, 23, 25, 27, 30, 31, 33, 36], moderate-dose atropine (0.01% to 0.5%) in four studies [13, 28, 29, 34], and high-dose atropine (1%) in five studies [15, 16, 24, 32, 35] together resulting in 21 interventional groups in 17 studies.

## 4. Methodology Quality Assessment

The quality of the included RCTs is shown in the supplementary materials (Figure S1). Overall, the trials seem to have a moderate risk of bias, with most of the trials reporting adequate random sequence generation, allocation concealment, and blinding of outcome assessment.

### 4.1. Efficacy Analysis

#### 4.1.1. Spherical Equivalent Refraction (SER)

**Figure 2:**
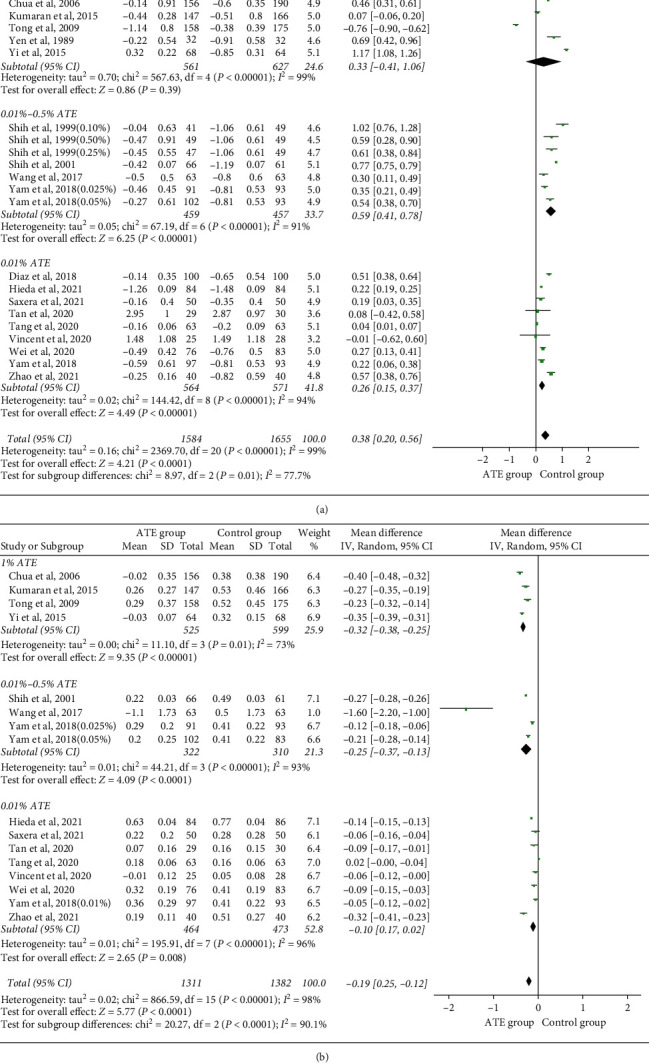
Forest plot of the effects of atropine on SER (a) and axial elongation (b). SER, spherical equivalent refraction; CI, confidence interval.

Data on annual rate of myopia progression were available from all studies. The progression of myopia was defined as the changes in SER relatively to baseline. The overall heterogeneity I^2^ was 99%, so a subgroup analysis was performed. The pooled data showed significantly progression for low dose (MD, 0.26 D per year; 95%CI, 0.15 to 0.37) and moderate dose (MD, 0.59 D per year; 95%CI, 0.41 to 0.78) compared to the control group. No statistically difference was found in high dose (*p*=0.32). There was a significant difference between the intervention and control group (*Z* = 4.26, *p* < 0.0001). The overall effect was 0.38 D per year (95% CI, 0.20 to 0.56), as shown in Figure 2(a).

#### 4.1.2. Axial Elongation

Sixteen trials from fourteen studies [13, 15, 16, 23, 24, 26, 27, 29, 34, 36] reported changes in axial length. The overall heterogeneity *I*^2^ was 98%, so a subgroup analysis was performed. The data showed significantly less axial elongation for low dose (MD, -0.10 mm per year; 95%CI, -0.17 to –0.02), moderate dose (MD, −0.25 mm per year; 95%CI, −0.37 to −0.13), and high dose (MD, −0.32 mm per year; 95%CI, −0.38 to −0.25) compared to the control group. The combined results demonstrated that atropine yields significantly greater improvement in myopia progression (*p* < 0.00001). The analyses reported that the overall MD was −0.19 mm per year (95% CI, −0.25 to −0.12), as shown in Figure 2(b).

#### 4.1.3. Rebound Effects

**Figure 3:**
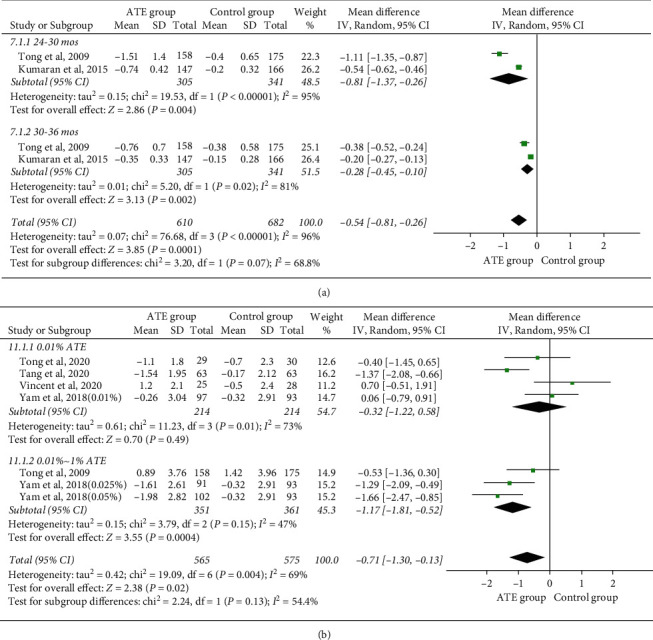
Forest plot of rebound effects (a) and AMP (b). AMP, accommodation amplitude; CI, confidence interval.

Four trials from two studies [16, 32] assessed the changes in SER. The overall heterogeneity I^2^ was 96%, so a subgroup analysis by variable of follow-up period was performed. The overall MD was −0.54 D per year (95%CI, −0.81 to −0.26) in high dose. Besides, there was significant difference between the first six months (MD, −0.81 D per year; 95%CI, −1.37 to −0.26) and the latter (MD, −0.28 D per year; 95%CI, −0.45 to −0.10) after discontinuation, indicating the annual rate of myopia progression was higher in short period of cessation (Figure 3(a)). There was a statistically significant difference that favored high-dose atropine (*p* < 0.00001).

#### 4.1.4. Adverse Effects

**Figure 4:**
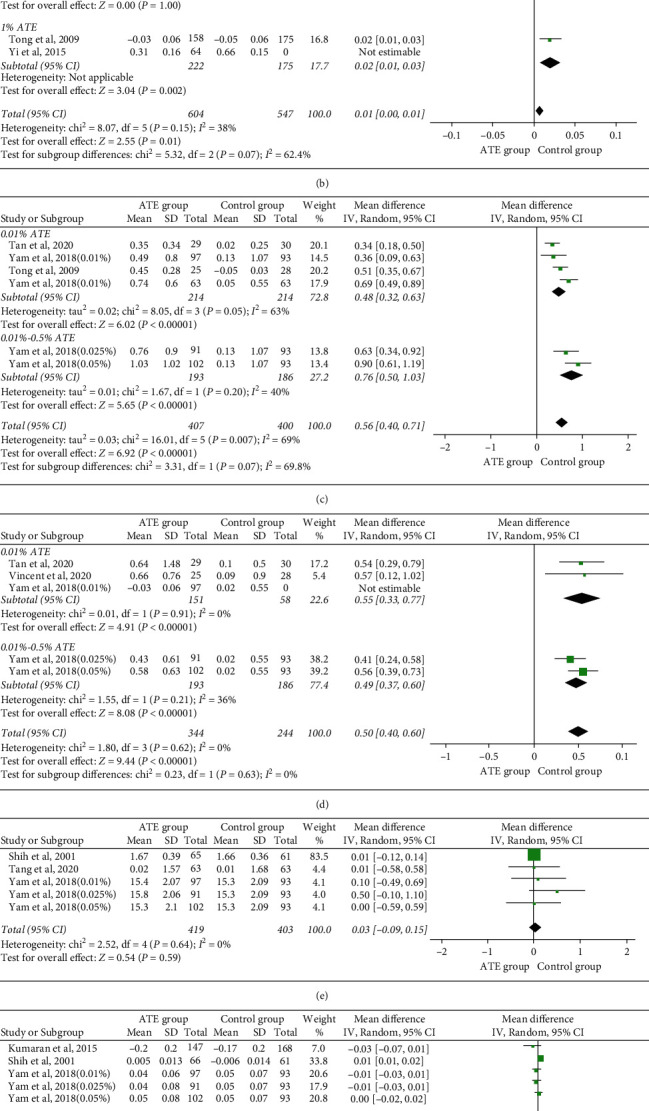
Changes of adverse effects of near vision (a), BCVA (b), photopic pupil size (c), mesopic pupil size (d), IOP (e), ACD (f), LT (g), and T-BUT (h). BCVA, best-corrected vision acuity; IOP, intraocular pressure; ACD, anterior chamber depth; LT, lens thickness; T-BUT, tear break-up time; and CI, confidence interval.

(1) Accommodation dysfunction: there were seven trials from five studies [13, 30–33] reporting data on changes of accommodation amplitude (AMP). The overall heterogeneity I^2^ was 69%, so a subgroup analysis was performed. The AMP was significantly reduced by 0.71 ± 0.58 D (*p*=0.02), respectively. Conversely, no statistically difference was identified in the low-dose atropine group (*p*=0.49, Figure 3(b)).

(2) Pupil size: we performed an analysis of changes in photopic [13, 30, 31, 33] and mesopic size [13, 30, 33] in six trials. The overall heterogeneity I^2^ was 69%, so a subgroup analysis was performed. There was a statistically significant difference in both factors (*p* < 0.00001). Low dose showed less influence on photopic pupil size than high dose (MD = 0.48 mm, 95%CI, 0.32 to 0.63), as shown in Figure 4 (*p*=0.07).

#### 4.1.5. BCVA

Seven trials from five studies [13, 15, 30–32] reported data in changes on BCVA. The overall heterogeneity *I*^2^ was 38%. No statistically significance was shown in low-dose atropine (MD = 0.01 log MAR, 95%CI, −0.00 to 0.01). The pooled data showed significance in the high-dose group (MD, 0.02 log MAR; 95% CI, 0.01 to 0.03), as shown in Figure 4.

#### 4.1.6. ACD, Near Vision, LT, IOP, and T-BUT

The overall heterogeneity I^2^ was 95% in LT and 65% in ACD. No heterogeneity was detected in near vision, IOP, and T-BUT. No statistically significant difference was found between the atropine and control groups in changes in ACD, near vision, LT, IOP, and T-BUT, as shown in Figure 4 (*p* > 0.05).

#### 4.1.7. Publication Bias

We performed a funnel plot using a random-effects model, and the effect size was *Z* = 4.26 (*p* < 0.0001). Publication bias may exist within the included studies (Figure S2).

#### 4.1.8. Sensitivity Analysis

We conducted a sensitivity analysis to determine the source of heterogeneity by removing studies one by one. We found limited difference between these trials, indicating that the results were relatively stable. A severe degree of heterogeneity was identified in subgroup differences in SER (*I*^2^ = 23.9%, *p*=0.27; *I*^2^ = 1.0%, *p*=0.36), as shown in Figure S3. The results of the sensitivity analysis demonstrated that one study [28], in which the inappropriate randomisation was found, had influenced the data analysis.

## 5. Discussion

### 5.1. Main Results

Our meta-analysis confirms that atropine is effective in slowing childhood myopic progression. There was a statistically significant difference among various doses of atropine. Low-dose atropine may be the most prominent dosage. This finding contrasts with a meta-analysis [20] published in 2017 that showed the same efficacy between various doses of atropine, but that analysis included RCTs and cohort studies together to investigate the overall effects of different doses. The previous meta-analysis [17] that included 11 studies and 1815 children and showed a positive effect of atropine, but no stratification by dose or quantification of adverse effects was performed and the 0.01% dose was not included. The next meta-analysis [37] included 7 studies and 1079 children and showed a positive effect of 0.01% atropine, but no significance in refraction values. Our study identified only high-quality RCTs and quantifies various doses of atropine. We have shown that decreasing the dose of atropine leads to a slowing myopic progression. Conversely, high-dose atropine had no influence on changes in SER, which could be explained by limited number of studies. We conclude that 1% atropine causes a rebound effect with more diopters in the former six months than the latter after discontinuation. The result was different from the study [19] by Chia et al. who identified the effectiveness of 0.01%, 0.1%, and 0.5% atropine in myopic children with no placebo control group, showing that 0.01% atropine rebounded less. This could be explained by quick and continual paralysis function of pupil dilation in the earlier period [38] that needs more RCTs to verify the rebound effects. We analyzed studies with the control group in rebound effects. For adverse effects, accommodation is an important factor in relation to atropine-induced side effects. In the current study, the MD change of −0.71 D in the atropine group was not significantly different from 0.26 D reported by Yam et al. [13] (*p*=0.142), but significantly lower than the 4.40 D found by Chia et al. [39] (*p*=0.0007). We confirm that high-dose atropine may alleviate AMP, but 0.01% atropine causes no effect on AMP. It may be the reason that high-dose atropine reduces compatible reaction, which may be a possible mechanism to prolong the development of myopia. However, these differences across studies may be related to different methodologies. Comparison of photopic pupil size between studies revealed that the MD increase in the current study was 0.48 mm (95% CI, 0.32 to 0.63) in the 0.01% atropine group, which was not significantly different from the finding of 0.49 mm in the study conducted by Yam et al. [13] (*p*=0.6045), but significantly smaller than the 0.91 mm observed by Chia et al. [19] (*p*=0.0014). The MD increase in mesopic pupil size was 0.49 mm (95%CI, 0.37 to 0.60) mm in 0.01%∼1% atropine groups in the current study, which was significantly higher than the 0.23 (0.46) mm in the study of Yam et al. [13] (*p*=0.0001), but significantly lower than the 1.15 (0.78) mm from Chia et al. [19] (*p*=0.0017). The small changes in pupil size noted in the present study may explain why subjects rarely complained regarding low-dose atropine use. We recommend using the lowest dose of atropine for therapy, and more clinical trials with doses are needed to investigate rebound effects in long-term application.

### 5.2. Strength and Limitations

To our best of knowledge, this is the first meta-analysis to systematically evaluate rebound effects of atropine and accommodation dysfunction. We included only high-quality RCTs [13, 15, 16, 23–36] providing evidence-based medical analysis for the use of atropine in controlling myopia. This meta-analysis verified that the effectiveness of atropine in controlling myopia progression was closely related to the dose. A 0.01% atropine might be the optimal dose which could slow the myopia progression and have no influence on accommodation. Meanwhile, the minimal additive effects in BCVA and photopic pupil size dilation were identified in low-dose atropine. Besides, 1.0% atropine had the least rebound effect after discontinuation, especially in the latter six months. The adverse effects of atropine in decreasing accommodation dysfunction were identified in high-dose atropine groups. Although atropine prevents myopia progression effectively, combined with other therapies, such as orthokeratology, and time spent outdoors, Tan et al. reported 0.01% atropine eye drops and orthokeratology can significantly slow the axis elongation compared to the use of orthokeratology alone [30]. Therefore, the effectiveness of the combined application of atropine and orthokeratology needed to be further studied. There were several limitations in our meta-analysis. First, although this meta-analysis had established strict inclusion and exclusion criteria, the heterogeneity was still high after using the subgroup analysis. Because not enough studies examined the rebound effect, different follow-up times of studies were combined in this meta-analysis to investigate the overall effects of different doses, which might be a source of additional heterogeneity. However, through the sensitivity analysis, the results of this meta-analysis were stable and consistent. Secondly, only data in rebound effects of high-dose atropine were available to analyze, but they lack of low-dose atropine measurement. The further determination and rebound effects of various doses required additional research, and more large-sample, multicenter, and high-quality RCTs will provide strong clinical evidence in the future.

## 6. Conclusions

In conclusion, the efficacy of atropine is closely associated with dose, and a rebound effect of high-dose atropine is more obvious in the former six months of cessation after discontinuation. The optical dose was reviewed as 0.01% atropine in the treatment of myopia and could be used as a clinically feasible method to control the progression of myopia with fewer accommodation dysfunction and adverse reactions.

## Figures and Tables

**Table 1:** Characteristics and demographics of included studies.

Author (y)	Country	Follow-up, mos	Intervention group	Control group	*N*	Age (y)	Baseline refraction
Chua et al., 2006 [24]	Singapore	24	1% ATE	Placebo	156/190	6∼12	−1.00 D to −6.00 D
Diaz-Llopis et al., 2018 [25]	Spain	60	0.01% ATE	Placebo	100/100	9∼12	−0.50 D to −2.00 D
Hieda et al., 2021 [26]	Japan	24	0.01% ATE	Placebo	85/86	6∼12	−1.00 D to −6.00 D
Kumaran et al., 2015 [16]	Singapore	36	1% ATE	Placebo	147/166	6∼12	−1.00 D to −6.00 D
Saxena et al., 2021 [27]	India	12	0.01%ATE	Placebo	50/50	6∼14	−0.5 D to −6.00 D
Shih et al., 1999 [28]	Taiwan	24	0.5%, 0.25%, 0.1% ATE	Tropicanide	137/49	6∼13	−0.5 D to −6.75 D
Shih et al., 2001 [29]	Taiwan	18	0.5% ATE + multifocal	Multifocal	66/61	6∼13	Mean, −3.28 D
Tan et al., 2020 [30]	Hong Kong	12	0.01% ATE + OK lens	OK lens	29/30	6∼11	−1.00 D to −4.00 D
Tang et al., 2020 [31]	China	12	0.01% ATE	Placebo	63/63	8∼14	−0.50 D to −6.00 D
Tong et al., 2009 [32]	Singapore	36	1% ATE	Placebo	158/175	6∼12	−1.00 D to −6.00 D
Vincent et al., 2020 [33]	Hong Kong	6	0.01% ATE + OK lens	OK lens	25/28	6∼11	−1.00 D to −4.00 D
Wang et al., 2017 [34]	China	12	0.5% ATE	Placebo	63/63	5∼10	−0.50 D to −2.00 D
Wei et al., 2020 [36]	China	12	0.01% ATE	Placebo	76/83	6∼12	−1.00 D to −6.00 D
Yam et al., 2018 [13]	Hong Kong	12	0.05%, 0.025%, 0.1% ATE	Placebo	290/93	4∼12	<−1.0 D D
Yen et al., 1989 [35]	Taiwan	12	1% ATE	Placebo	32/32	6∼14	−0.5 D to −4.00 D
Yi et al., 2015 [15]	China	12	1% ATE	Placebo	68/64	7∼12	−0.50 D to −2.00 D
Zhao and Hao, 2021 [23]	China	12	0.01% ATE + OK lens or spectacles	OK lens or spectacles	40/40	5∼14	−1.00 D to −6.00 D

Y, year; mos, months; N, number; ATE, atropine; OK lens: orthokeratology lens; D, diopters; -, none.

## Data Availability

All data relevant to the study are included in the article or uploaded as online supplementary information.

## Supplementary Materials

Supplementary Materials FigS1. Method quality of evaluation. Figure S2. Funnel plot of the subgroups. SE, standard deviation. MD, mean difference. SER (A), axial elongation (B), AMP (C), photopic pupil size (D), ACD (E), and total (F). SER, spherical equivalent refraction; AMP, accommodation amplitude; ACD, anterior chamber depth; SE, standard deviation. MD, mean difference; ATE, atropine. Figure S3. Sensitivity Analysis of subgroup differences in SER (A), axial elongation (B), AMP (C), photopic pupil size (D), and ACD (E). SER, spherical equivalent refraction; AMP, accommodation amplitude; ACD, anterior chamber depth; and CI, confidence interval.
