# Supplementary material for: Efficacy and Adverse Effects of Atropine for Myopia Control in Children: A Meta-Analysis of Randomised Controlled Trials
Source: J Ophthalmol. 2021 Dec 10;2021:4274572. doi: 10.1155/2021/4274572 (PMC8683246; doi:10.1155/2021/4274572)
Supplement: Supplementary Materials — FigS1. Method quality of evaluation. Figure S2. Funnel plot of the subgroups. SE, standard deviation. MD, mean difference. SER (A), axial elongation (B), AMP (C), photopic pupil size (D), ACD (E), and total (F). SER, spherical equivalent refraction; AMP, accommodation amplitude; ACD, anterior chamber depth; SE, standard deviation. MD, mean difference; ATE, atropine. Figure S3. Sensitivity Analysis of subgroup differences in SER (A), axial elongation (B), AMP (C), photopic pupil size (D), and ACD (E). SER, spherical equivalent refraction; AMP, accommodation amplitude; ACD, anterior chamber depth; and CI, confidence interval. [file 4274572.f1.zip › 4274572.f1/Figures S2 and S3.docx]

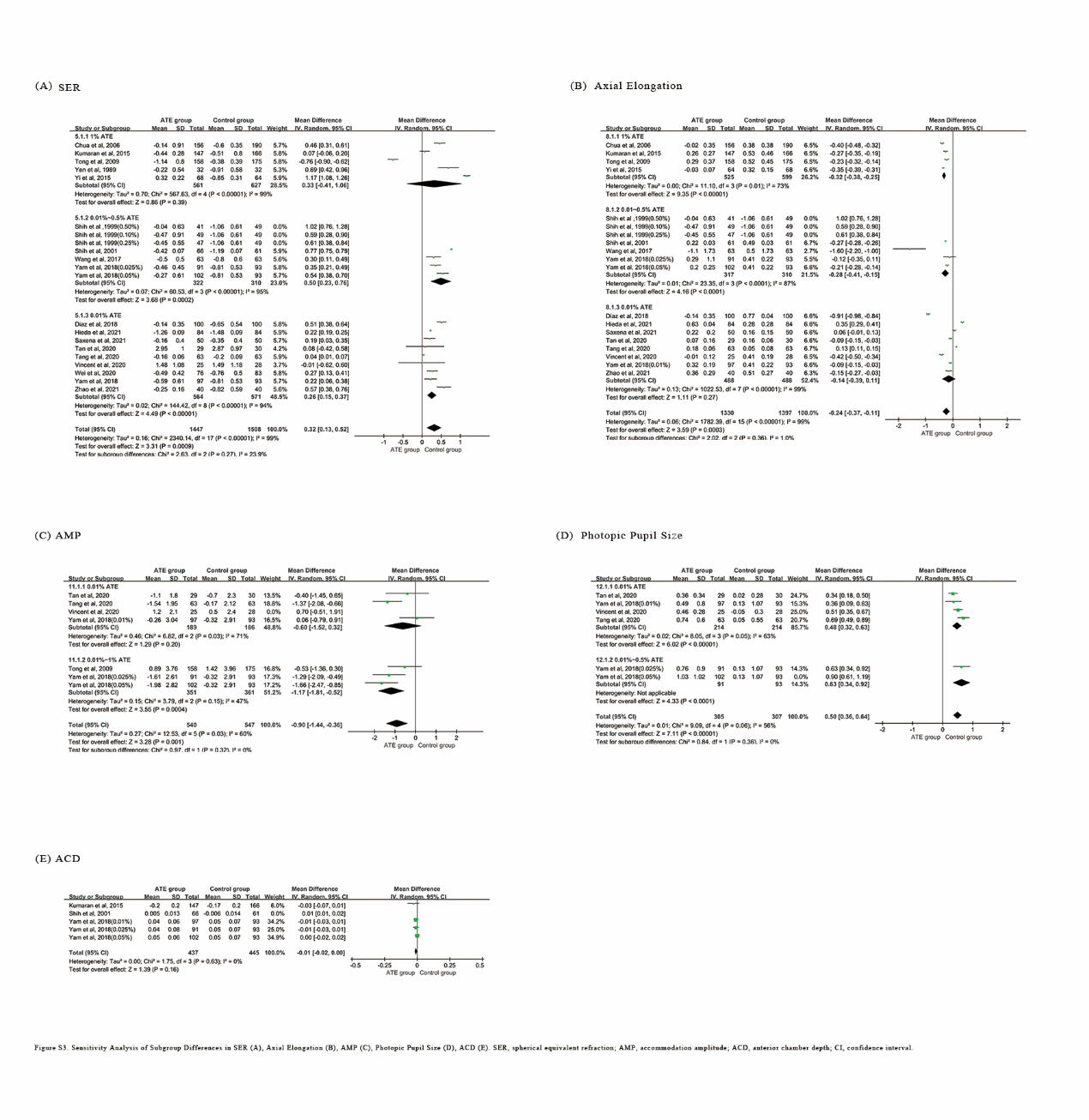


Figure S3. Sensitivity Analysis of Subgroup Differences in SER (A), Axial Elongation (B), AMP (C), Photopic Pupil Size (D), ACD (E). SER, spherical equivalent refraction; AMP, accommodation amplitude; ACD, anterior chamber depth; CI, confidence interval. Publication bias was analyzed in the optical studies and subgroups as shown in Figure S2.


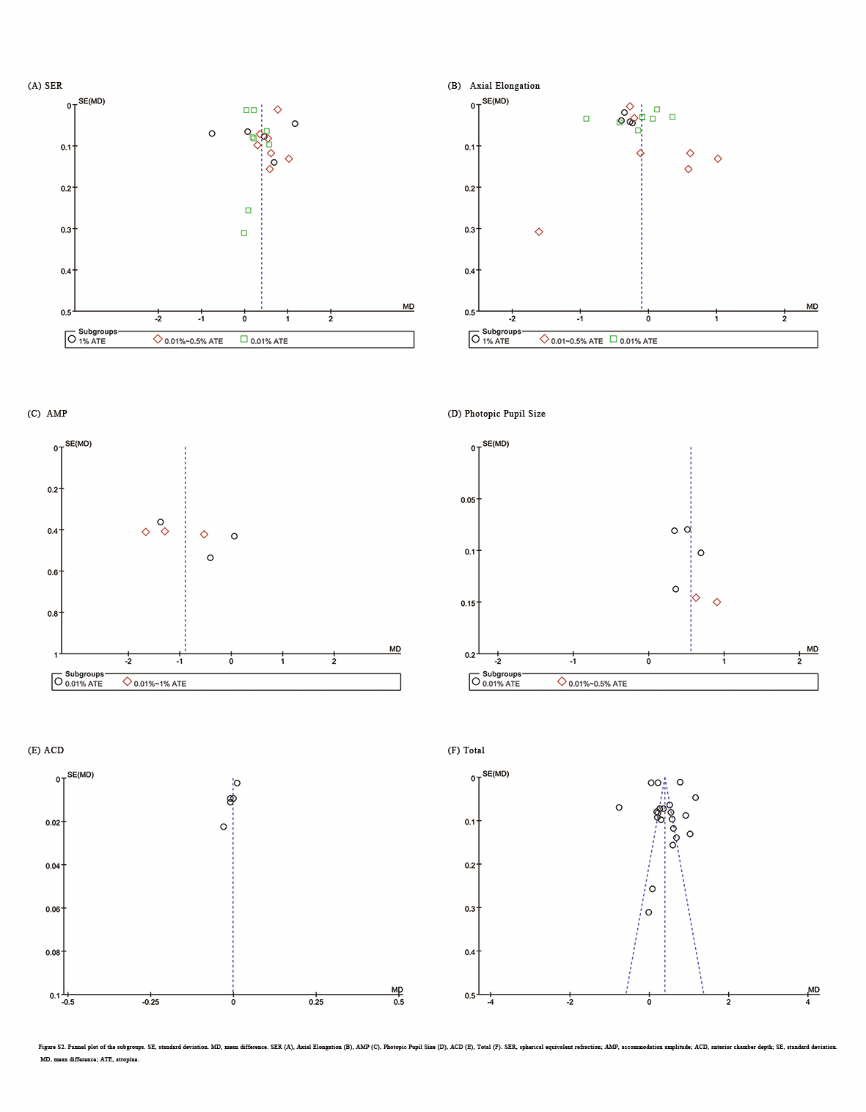


Figure S2. Funnel plot of the subgroups. SE, standard deviation. MD, mean difference. SER (A), Axial Elongation (B), AMP (C), Photopic Pupil Size (D), ACD (E), Total (F). SER, spherical equivalent refraction; AMP, accommodation amplitude; ACD, anterior chamber depth; SE, standard deviation. MD, mean difference; ATE, atropine.
